# Psychometric Properties of Caregiver Contribution and Self‐Efficacy Scales in Cancer

**DOI:** 10.1111/nhs.70380

**Published:** 2026-06-30

**Authors:** Marco Di Nitto, Federica Lacarbonara, Francesco Torino, Tatiana Bolgeo, Vincenzo Damico, Greta Ghizzardi, Sipontina Rita Zerulo, Rosaria Alvaro, Angela Durante, Paolo Iovino, Ercole Vellone

**Affiliations:** ^1^ Department of Health Sciences University of Genoa Genoa Italy; ^2^ Azienda Ospedaliera—University of Padova Padua Italy; ^3^ Department of Systems Medicine, Medical Oncology Tor Vergata University of Rome Rome Italy; ^4^ Research Training Innovation Infrastructure—Department of Research and Innovation Azienda Ospedaliero Universitaria SS Antonio e Biagio e Cesare Arrigo Alessandria Italy; ^5^ Azienda Socio‐Territoriale di Lecco Lecco Italy; ^6^ School of Nursing, Directorate of Nursing and Allied Health Professions Azienda Socio‐Territoriale di Lodi Lodi Italy; ^7^ Azienda Ospedaliera Universitaria Policlinico Riuniti di Foggia Foggia Italy; ^8^ Department of Biomedicine and Prevention Tor Vergata University of Rome Rome Italy; ^9^ Sant'Anna School of Advanced Studies, Health Science Interdisciplinary Center Pisa Italy; ^10^ Fondazione Toscana “Gabriele Monasterio” Pisa Italy; ^11^ Department of Health Sciences University of Florence Florence Italy; ^12^ Department of Nursing and Obstetrics Wroclaw Medical University Wroclaw Poland

**Keywords:** chronic disease management, family nursing, instrument reliability, instrument validation, neoplasms, oncology nursing

## Abstract

This study aimed to validate the Caregiver Contribution to Self‐Care of Chronic Illness Inventory (CC‐SC‐CII) and the Caregiver Self‐Efficacy in Contributing to Patient Self‐Care Scale (CSE‐CSC) in caregivers of patients with cancer. A multicenter, cross‐sectional validation study was conducted involving a convenience sample of caregivers recruited from five Italian oncology centers. Confirmatory factor analysis (CFA) was used to assess structural validity, internal consistency was evaluated using factor score determinacy and McDonald's Omega, and construct validity was examined through correlations between caregiver and patient self‐care scores. Test–retest reliability was assessed using the intraclass correlation coefficient. A total of 412 caregivers were included. CFA supported the three‐factor structure of the CC‐SC‐CII and the bidimensional structure of the CSE‐CSC, with adequate fit indices for all scales. Internal consistency was excellent across subscales. Construct validity was supported by positive associations between caregiver self‐efficacy and caregiver contribution dimensions, while inverse associations emerged with patient self‐care in selected domains. This study contributes to the limited evidence on caregiver support to self‐care in cancer, offering validated tools to guide clinical assessment and research on this topic.

## Key Points

- What this paper adds
  ○ This study provides the first validation of two caregiver self‐care instruments in caregivers of patients with cancer.
  ○ The findings confirm the robust factorial structure, reliability, and validity of both scales in the oncology context.
  ○ The results reveal domain‐specific and sometimes attenuated associations between caregiver contribution and patient self‐care, reflecting the complex and shared dynamics of caregiving in oncology.

## 1 Background

Caregiver contribution to self‐care (CC to self‐care) refers to a set of caregiver behaviors intended to support and assist the patient in carrying out self‐care activities (e.g., encouraging physical activity) (Vellone et al. 2019). CC to self‐care is conceptualized as comprising three components: contribution to self‐care maintenance, which involves actions aimed at maintaining the stability of the chronic condition (e.g., supporting adherence to treatment, facilitating daily routines, or dietary management); contribution to self‐care monitoring, which includes behaviors related to observing and tracking signs and symptoms of disease exacerbation (e.g., monitoring side effects of treatment); and contribution to self‐care management, which encompasses caregiver involvement in addressing symptom exacerbation and managing medication‐related complications (e.g., handling treatment side effects or acute situations) (Sollazzo et al. 2023). To date, CC to self‐care has been studied in caregivers of patients with multiple chronic conditions, as well as those caring for individuals with specific diseases such as heart failure, chronic obstructive pulmonary disease COPD, and diabetes. Adequate CC can effectively improve patients outcomes. In a previous study that involved patients with heart failure and their caregivers, higher CC was associated with higher patient's quality of life (Caggianelli et al. 2024). However, a recent comparative study (Erba et al. 2025) found that caregivers often face difficulties in adequately performing CC to self‐care activities, tending to focus more on following provider prescriptions than on supporting lifestyle changes.

Another construct that has been extensively explored in self‐care research is self‐efficacy, recognized as a key mediator of patient self‐care (Riegel et al. 2012). Self‐efficacy has also been studied in caregivers, specifically referring to the caregiver's confidence in their ability to support the patient's self‐care (Vellone et al. 2019). Caregiver self‐efficacy has been positively associated with greater caregiver involvement in self‐care behaviors and with higher levels of patient self‐care across several chronic illness populations (Lee et al. 2025), and it has been investigated in caregivers of patients with multiple chronic conditions (De Maria et al. 2021), as well as in those caring for patients with COPD (Matarese et al. 2023) and heart failure (Vellone, Biagioli, et al. 2020), where its mediating role between predictors of CC to self‐care and CC to self‐care itself has been demonstrated.

Cancer is a compelling example of why CC to self‐care is needed, as its management requires complex and sustained self‐care behaviors. Beyond the well‐documented physical, psychosocial, relational, and economic burden experienced by caregivers (Bradley 2019), cancer frequently co‐occurs with other chronic health conditions or leads to long‐term treatment‐related sequelae that require ongoing management (Ahmad et al. 2023). In this context, self‐care involves not only coping with symptom burden and multimorbidity, but also performing concrete daily behaviors such as medication administration, adherence monitoring, recognition and management of treatment‐related toxicities, prevention of complications, and timely decision‐making regarding when to seek professional support. These demands are particularly important for patients receiving oral anticancer agents (OAAs), for whom treatment management largely occurs at home and is often supported by informal caregivers (Lv et al. 2023). Assessing CC to self‐care and caregiver self‐efficacy is therefore essential to capture caregivers' role in supporting these self‐care behaviors.

To promote the shift from hospital to home‐centered care and ensure adequate management of patients with chronic conditions, it is essential to accurately measure CC to self‐care and self‐efficacy using tools that are reliably applicable across different chronic diseases. This approach would enable not only a comprehensive assessment of patients with multimorbidity using a single instrument, but also an optimized nursing evaluation focused on the full range of self‐care behaviors. However, although both the Caregiver Contribution to Self‐Care of Chronic Illness Inventory (CC‐SC‐CII) and the Caregiver Self‐Efficacy in Contributing to Patient Self‐Care Scale (CSE‐CSC) have demonstrated solid psychometric properties in caregivers of individuals with various chronic illnesses, their measurement properties have not yet been evaluated in the context of cancer care. Indeed, in previous studies where these two instruments have been used, patients with cancer were not considered or included (Adëraj et al. 2025; Erba et al. 2025). The CC‐SC‐CII and the CSE‐CSC were selected because, as reported above, they are widely used instruments within the self‐care of chronic illness framework and have demonstrated good psychometric properties across different chronic disease populations, allowing comparisons across conditions. Therefore, the aim of this study was to test the psychometric properties of the CC‐SC‐CII and the CSE‐CSC in patients living with cancer.

## 2 Methods

### 2.1 Design

This study is part of an ongoing longitudinal research project investigating self‐care behaviors in patients receiving OAAs. The protocol of the longitudinal research project is available in Open Science Framework (osf.io/arw82). The present manuscript adheres to the COnsensus‐based Standards for the Selection of Health Measurement INstruments (COSMIN) guidelines (Gagnier et al. 2021) (Supporting Information S1).

### 2.2 Setting and Sample

A convenience‐based consecutive sample of caregivers of individuals receiving OAAs was recruited from five outpatient facilities (hospital‐based and community‐based clinics) in Italy (Alessandria, Lodi, Lecco, Rome, and Foggia) between November 2022 and April 2024. The study included informal caregivers, defined according to Castro et al. as: “*Individuals, often family members or friends, who provide ongoing care, typically at home, to chronic patients with varying degrees of disability*” (Castro et al. 2023).

Caregivers were eligible for inclusion if they met the following criteria: (1) aged 18 years or older; (2) provided written informed consent to participate in the study; (3) were caring for a patient diagnosed with metastatic or locally advanced solid cancer who had been actively receiving OAAs (including cytotoxic chemotherapy, molecular targeted therapy, or hormone therapy) for at least 3 months; (4) were oriented to people and places; and (5) were able to read and understand the Italian language. Participants were excluded if they were paid caregivers. Eligible participants were provided with detailed information about the study and invited to take part.

The sample size estimation followed a commonly accepted rule of thumb, requiring a minimum of 200 individuals, which is considered sufficient for effective confirmatory factor analysis (CFA) (Kline 2023).

### 2.3 Instruments

The CC‐SC‐CII Version 2 was used to measure CC to self‐care behaviors in chronic conditions (Vellone, Lorini, et al. 2020). This 19‐item instrument consists of three separate scales assessing: (1) CC to self‐care maintenance (i.e., how often, during the last month, a caregiver recommended that the care recipient adopt behaviors aimed at maintaining physical and mental stability of a chronic illness); (2) CC to self‐care monitoring (i.e., how often a caregiver recommended that the care recipient monitor signs and symptoms of their chronic illness), and; (3) CC to self‐care management (i.e., how often a caregiver contributed to symptom recognition/interpretation and responded to symptoms of chronic illness exacerbation). The maintenance, monitoring, and management scales consist of 7, 5, and 7 items, respectively. Each item of the CC‐SC‐CII is rated on a 5‐point Likert scale ranging from 1 (“never”) to 5 (“always”). The CC to self‐care management scale can only be completed if the patient reported to their caregiver the occurrence of any clinical symptom due to the chronic illness during the last month. Each scale yields a score ranging from 0 and 100, with higher scores indicating better CC to self‐care. Scores ≥ 70 indicate adequate CC to self‐care. In the original validation study, the CC‐SC‐CII was tested for structural and concurrent validity, demonstrated a stable three‐factor structure consistent with the self‐care framework and satisfactory internal consistency across the maintenance (*α* = 0.833), monitoring (*α* = 0.926), and management (*α* = 0.761) scales (Vellone, Lorini, et al. 2020).

The Self‐Care of Chronic Illness Inventory (SC‐CII) is a generic measure of self‐care in chronic illness. It has been developed based on the Middle Range Theory of Self‐care of Chronic Illness and it is characterized by the same three dimensions, item distribution, Likert type, score calculation and interpretation of the CC‐SC‐CII. However, the SC‐CII asks patients which behaviors they put in place to maintain, monitor and manage the chronic disease. This instrument has been recently validated in the cancer population (Di Nitto et al. 2025). In the latter study, the SC‐CII was tested for structural and concurrent validity, demonstrated a stable three‐factor structure consistent with the self‐care framework and satisfactory internal consistency across the maintenance (ωtotal = 0.89)., monitoring (*α* = 0.97), and management (ωtotal = 0.96) scales.

The CSE‐CSC Scale is a 10‐item instrument developed to measure caregiver self‐efficacy in supporting patient self‐care maintenance, monitoring, and management of chronic illness (De Maria et al. 2021). Caregivers are asked to report the extent to which they feel confident in helping the patients they care for to perform self‐care activities. Each item of the CSE‐CSC is rated on a 5‐point Likert scale ranging from 1 (“not confident”) to 5 (“very confident”). The total score ranges from 0 to 100, where higher scores indicate greater caregiver self‐efficacy in contributing to the self‐care of a patient with chronic illness. The CSE‐CSC was tested for structural and concurrent validity, demonstrated a stable two‐factor structure consistent with the self‐care framework, and showed excellent internal consistency (*α* = 0.942) (De Maria et al. 2021).

We also used a sociodemographic questionnaire, developed by the research team, to collect patients' and caregivers' sociodemographic information (e.g., age, gender, time dedicated to caregiving).

### 2.4 Data Collection

Trained nurse research assistants screened inpatients and outpatients from participating centers to identify eligible participants based on predefined inclusion and exclusion criteria. Those who met the eligibility criteria received detailed information about the study and were invited to participate. Data collection commenced only after participants had signed the informed consent form. In this project, CC‐SC‐CII and CSE‐CSC were administered at the time of caregiver enrolment and again 3 months later, while patients' Self Care‐ chronic illness inventory (SC‐CII) and patients' Self‐Care Self‐Efficacy Scale (SE‐SC) were collected 3 months after patient enrolment. The three‐month interval between test and retest was selected because it reflects the standard time interval between consecutive follow‐up visits for patients receiving oral anticancer agents, allowing assessment of instrument stability over a clinically meaningful period of home‐based care.

### 2.5 Ethical Considerations

This study adhered to the Good Clinical Practice Standards of the European Union and the Helsinki Declaration. Before starting data collection, this study was approved by the Ethics Committee of the “Policlinico Tor Vergata of Rome” on 20/09/2022 (reference number 188.22). Participation was voluntary; all participants were fully informed about the study aims and could withdraw from the study at any time. Enrolment occurred only after participants had signed the informed consent form.

### 2.6 Statistical Analysis

Descriptive statistics, including means, standard deviations, and proportions, were used to characterize the sample and summarize the responses to the CC‐SC‐CII and CSE‐CSC scales. Floor and ceiling effects were examined separately at the item and scale‐score levels. At the item level, floor and ceiling effects were considered present when more than 50% of respondents endorsed the lowest or highest response category, respectively (Polit and Yang 2016). At the scale‐score level, floor and ceiling effects were considered present when more than 15% of respondents achieved the lowest or highest possible score, respectively (Terwee et al. 2007). Inter‐item correlations and corrected item‐total correlations were also calculated for each scale or subscale. Inter‐item correlations between 0.30 and 0.70 were considered appropriate, whereas corrected item‐total correlations > 0.30 were considered acceptable (Polit and Yang 2016).

Consistent with classical test theory, a confirmatory factor analytical approach (CFA) was conducted to investigate the structural validity of the scales. The factor structure of the CC‐SC‐CII (two‐factors model for maintenance, one‐factor model for monitoring and two‐factors model for management) and CSE‐CSC (one‐factor model) scales was derived from the corresponding patient versions, which have been recently validated in the cancer population (Di Nitto et al. 2025). Anticipating a possible misfit of these models, we also considered the factorial solutions from previous Italian validations (De Maria et al. 2021; Vellone, Lorini, et al. 2020).

Multivariate normality was assessed using Mardia's test (Korkmaz et al. 2014), which indicated a significant deviation from normality across all scales. Therefore, a robust maximum likelihood estimator was used to obtain parameter estimates, standard errors, and fit indices that are less sensitive to violations of the normality assumption. We examined model fit by means of the following fit indices: *χ* ^2^ statistics, Comparative Fit Index (CFI: ≥ 0.90 acceptable fit, CFI: ≥ 0.95 optimal fit), Tucker and Lewis Index (TLI: ≥ 0.90 acceptable fit, TLI: ≥ 0.95 optimal fit), Root Mean Square Error or Approximation (RMSEA: ≤ 0.08 acceptable fit), and Standardized Root Mean Square Residual (SRMR: ≤ 0.08 acceptable fit) (Hu and Bentler 1999). The *χ* ^2^ statistic was reported for completeness but not interpreted because this index is excessively sensitive to sample size (Alavi et al. 2020). Consistent with prior studies (Adëraj et al. 2025; De Maria et al. 2021; Di Nitto et al. 2025), each scale was tested separately, with one CFA performed for each scale. Modification indices were inspected to identify potential areas of model misfit. Residual covariances were considered only when the modification index exceeded the conventional threshold of 3.84 (corresponding to a *χ* ^2^ improvement with 1 degree of freedom at *p* < 0.05) and when a clear theoretical rationale existed (Brown 2015; Kline 2023). When high correlations between first‐order factors were observed, higher‐order factor models were specified to represent the shared variance underlying the lower‐order dimensions. Thus, the higher‐order models were used to examine whether the first‐order behavioral facets could be represented by a common higher‐order construct, rather than to replace the interpretation of the first‐order factors. Higher‐order model was tested because the Middle‐Range Theory of Self‐Care of Chronic Illness conceptualizes self‐care maintenance, monitoring, and management as broader self‐care processes, and first‐order factors represent behavioral facets within those broader processes (Riegel et al. 2012). In cases where the higher‐order factor was defined by only two first‐order factors, model identification was achieved by fixing the variance of the second‐order factor to 1.0 (Brown 2015). No additional constraints, such as equality constraints on factor loadings or residual variances, were imposed beyond fixing the second‐order factor variance to 1.0.

Internal consistency was estimated with the factor score determinacy (Muthén and Muthén 2017) and Omega coefficient, whose values should be ≥ 0.70 to indicate adequate internal consistency (McDonald 1999). To assess the stability of the CC‐SC‐CII and CSE‐CSC, test–retest reliability was evaluated by readministering the instruments to a subsample of patients with stable cancer. This form of reliability was assessed with the intraclass correlation coefficient (ICC), calculated using two‐way random effects models on the scores of each scale and subscale. An ICC ≥ 0.70 was considered adequate (Kline 2023).

Construct validity was assessed via hypothesis testing. First, we hypothesized a positive and moderate correlation between CC to self‐care and patients' self‐care, measured using the SC‐CII as hypothesized in a previous study (Vellone, Lorini, et al. 2020), based on the assumption that greater caregiver involvement supports patients' self‐care behaviors. Second, we hypothesized moderate positive correlations between the three subscales of the CC‐SC‐CII and CSE‐CSC, in accordance with self‐efficacy theory (Vellone et al. 2019) and previous validation studies (De Maria et al. 2021; Vellone, Lorini, et al. 2020). Pearson's correlation coefficients were used exclusively for construct validity hypothesis testing and not for the assessment of reliability over time. Correlation magnitudes were interpreted as small (0.10–0.29), moderate (0.30–0.49), and strong (≥ 0.50).

For all analyses, the level of significance was set at *p* ≤ 0.05 (two‐tailed). Since the missing data in SC‐CII items were less than 5%, only complete cases were used for analysis. Descriptive statistics and correlations were performed using R (version 4.4.2) along with the “*table1*”, “*correlation*” and “*irr*” packages, while Mplus v.8.9 (Muthén and Muthén, Los Angeles, CA) was used to conduct the CFA.

## 3 Results

### 3.1 Descriptives of the Sample

In total, 412 caregivers and their respective care recipients were included in the analysis. Regarding caregivers' characteristics, the majority were female (*n* = 258, 62.62%) and aged between 18 and 50 years (*n* = 230, 55.83%). Most participants were married (*n* = 302, 73.30%), were employed (*n* = 236, 57.28%), and all caregivers reported cohabiting at least with another person. Regarding caregiving responsibilities, most participants lived with their care recipient (*n* = 273, 66.26%), and the primary relationship with the care recipient was spouse (*n* = 218, 52.91%). Over half of the participants provided up to 10 h of caregiving per week (*n* = 227, 55.10%), and they reported being caregivers for a mean of 40.03 months (±84.18) (Table 1).

**TABLE 1:** Sociodemographic and clinical characteristics of the sample (*n* = 412).

	*N* (%)	Mean (SD)	Missing (%)
Gender			1 (0.24)
Male	153 (37.14)		
Female	258 (62.62)		
Age			3 (0.73)
18 to 50	230 (55.83)		
51 to 65	118 (28.64)		
66 to 75	47 (11.41)		
≥ 76	14 (3.40)		
Marital status			1 (0.24)
Single	91 (22.09)		
Married	302 (73.30)		
Divorced, separated	11 (2.67)		
Widowed	7 (1.70)		
Education			2 (0.49)
Elementary school	9 (2.18)		
Secondary school	73 (17.72)		
High school	206 (50.00)		
University degree	122 (29.61)		
Employment status			1 (0.24)
Unemployed/retired/homemaker	87 (21.12)		
Freelance	56 (13.59)		
Employed	236 (57.28)		
Student	24 (5.83)		
Other	8 (1.94)		
Income			1 (0.24)
Insufficient	21 (5.10)		
Sufficient	126 (30.58)		
Good	257 (62.38)		
Excellent	7 (1.70)		
Household size			25 (6.07)
Lives with one person	132 (32.04)		
Lives with two persons	84 (20.39)		
Lives with > 2 persons	171 (41.50)		
Do you live with your care recipient			1 (0.24)
Yes	273 (66.26)		
No	138 (33.50)		
Relationship with care recipient			2 (0.49)
Spouse	218 (52.91)		
Son	121 (29.37)		
Sibling	11 (2.67)		
Nephew/Niece	26 (6.31)		
Other	13 (3.16)		
No relationship	21 (5.10)		
Weekly hours of caregiving			35 (8.50)
0 to 10	227 (55.10)		
11 to 20	43 (10.44)		
21 to 30	48 (11.65)		
≥ 30	59 (14.32)		
Months since caregiving began		40.03 (±84.18)	26 (6.31)
0 to 12	221 (53.64)		
13 to 24	50 (12.14)		
24 to 48	43 (10.44)		
≥ 49	73 (17.72)		

Regarding patients' characteristics, the sample included 228 females (55.34%), most of the sample had an age < 66 years (*n* = 262, 63.59%), a high‐school education or higher title (*n* = 269, 65.29%), and had a relationship (*n* = 312, 75.73%). Clinically, the most frequent tumor sites were genitourinary (*n* = 125, 30.34%) and breast (*n* = 126, 30.58%) (Table 2).

**TABLE 2:** Sociodemographic and clinical characteristics of the care recipients (*n* = 412).

	*N* (%)	Missing (%)
Sex		0 (0)
Male	228 (55.34)	
Female	184 (44.66)	
Age		2 (0.49)
18 to 50	100 (24.27)	
51 to 65	162 (39.32)	
66 to 75	90 (21.84)	
	58 (14.08)	
Marital status		1 (0.24)
Single	99 (24.03)	
In a relationship	312 (75.73)	
Education		2 (0.49)
None or Elementary	49 (11.89)	
Secondary	92 (22.33)	
High school	196 (47.57)	
University	73 (17.72)	
Tumor site		2 (0.49)
Lung	53 (12.86)	
Genitourinary	125 (30.34)	
Breast	126 (30.58)	
Other	65 (15.78)	
Gastrointestinal	41 (9.95)	

### 3.2 Descriptives of the Responses to the Items and Item‐Total Correlations

Table 3 shows the descriptives of the items of the CC‐SC‐CII and CSE‐CSC. Self‐care maintenance, monitoring and management and self‐efficacy mean scores were all high and above the cut‐off of 70 (respectively 77.77, 88.29, 78.55 and 75.54) (Table 2). At the item level, no floor effects were observed for either instrument, as no item had more than 50% of respondents endorsing the lowest response category. Conversely, high endorsement of the highest response category was common for the CC‐SC‐CII. Using the 50% item‐level threshold, 17 CC‐SC‐CII items showed a ceiling effect, with the highest proportions observed for item 13 (76.0%). In contrast, no CSE‐CSC item exceeded the 50% threshold for item‐level ceiling effects. At the scale‐score level, using the conventional 15% threshold, no floor effects were observed for the CC‐SC‐CII maintenance, monitoring, and management scales, or for the CSE‐CSC total score (1.47%, 0.49%, 0.49%, and 0.24%, respectively). Ceiling effects were observed for the CC‐SC‐CII maintenance (18.09%), monitoring (58.92%), and management (23.77%) scales, whereas the CSE‐CSC total score did not exceed the 15% threshold (14.18%). The items of the CC‐SC‐CII with the highest score for self‐care maintenance, monitoring and management were respectively item #2 (“Try to avoid getting sick”), item #9 (“Pay attention to changes in how one feels”) and item #17 (“Tell about the symptom to the healthcare provider of the person you care for at the next office visit”) while the items with the lowest score were respectively item #1 (“Make sure to get enough sleep”), item #10 (“Monitor for medication side‐effects”) and item #19 (“Think of a treatment you used the last time the person you care for had symptoms. Did the treatment you used make him/her feel better”). For the CSE‐CSC the item with the highest score was item #3 (“Persist in following the treatment for the person you care for even when difficult”), while the item with the lowest score was item #1 (“Keep the person you care for stable and free of symptoms”).

**TABLE 3:** Descriptives and item total correlations of the items of the CC‐SC‐CII and CSE‐CSC.

CC‐SC‐CII	Mean (SD)	Sk	Ku	ITC
CC‐ to self‐care maintenance (total score, 0–100)	77.77 (25.58)	−1.21	0.57	—
Health‐promoting behaviors				
1. Make sure to get enough sleep?	3.95 (1.41)	−1.02	−0.42	0.78
2. Try to avoid getting sick (e.g., flu shot, wash your hands)?	4.35 (1.17)	−1.76	1.87	0.76
3. Do physical activity (e.g., take a brisk walk, use the stairs)?	3.99 (1.34)	−1.05	−0.23	0.81
7. Do something to relieve stress (e.g., mindfulness, yoga, music)?	3.98 (1.12)	−1.13	0.63	0.57
Illness‐related behaviors				
4. Eat special foods or avoid certain foods?	4.00 (1.37)	−1.11	−0.13	0.73
5. Keep appointments for routine or regular health care?	4.25 (1.26)	−1.58	1.20	0.77
6. Take prescribed medicines without missing a dose?	4.28 (1.21)	−1.53	1.12	0.65
CC‐ to self‐care monitoring (total score, 0–100)	88.29 (19.37)	−1.94	3.53	
8. Monitor the health condition?	4.55 (0.90)	−2.11	3.89	0.78
9. Pay attention to changes in how one feels?	4.57 (0.79)	−2.02	3.74	0.84
10. Monitor for medication side‐effects?	4.45 (0.96)	−1.89	2.99	0.80
11. Monitor whether one tires more than usual doing normal activities?	4.56 (0.80)	−1.92	3.36	0.83
12. Monitor for symptoms?	4.54 (0.98)	−2.34	4.82	0.74
CC to self‐care management (total score, 0–100)	78.55 (25.24)	−1.20	0.41	
Autonomous behaviors				
14. Change what the person you care for eats or drinks to make the symptom decrease or go away?	4.16 (1.17)	−1.25	0.53	0.82
15. Recommend the person you care for to change the activity level (e.g., slow down, rest)?	4.23 (1.10)	−1.28	0.68	0.83
Consulting behaviors				
16. Recommend the person you care for to take a medicine to make the symptom decrease or go away?	4.16 (1.24)	−1.37	0.72	0.79
17. Tell about the symptom to the healthcare provider of the person you care for at the next office visit?	4.39 (1.09)	−1.79	2.25	0.76
18. Call the healthcare provider of the person you care for to get guidance?	4.35 (1.09)	−1.78	2.71	0.68
19. Think of a treatment you used the last time the person you care for had symptoms. Did the treatment you used make him/her feel better?	3.85 (1.22)	−0.91	−0.03	0.59
CSE‐CSC (total score, 0–100)	75.54 (18.63)	−0.66	0.21	
Self‐efficacy in self‐care maintenance and monitoring				
1. Keep the person you care for stable and free of symptoms?	3.66 (1.04)	−0.81	0.40	0.51
2. Follow the treatment advice have been given to the person you care for?	4.16 (0.82)	−0.89	0.72	0.64
3. Persist in following the treatment for the person you care for even when difficult?	4.20 (0.82)	−0.95	0.95	0.73
4. Monitor the health condition of the person you care for routinely?	4.14 (0.88)	−0.94	0.66	0.75
5. Persist in routinely monitoring the health condition of the person you care for even when difficult?	4.05 (0.98)	−0.89	0.24	0.79
Self‐efficacy in self‐care management				
6. Recognize changes in health of the person you care for if they occur?	4.02 (0.97)	−0.73	0.25	0.82
7. Evaluate the importance of the symptoms of the person you care for?	4.11 (0.98)	−1.01	0.45	0.77
8. Do something to relieve the symptoms?	3.94 (1.02)	−0.74	−0.18	0.82
9. Persist in finding a remedy for the symptoms even when difficult?	3.99 (0.99)	−0.80	−0.08	0.79
10. Evaluate how well a remedy works?	3.97 (0.93)	−0.93	0.84	0.75

*Note:* Total scores for each scale of the CC‐SC‐CII and CSE‐CSC range from 0–100. All items are scored on a 5‐point Likert scale.

Abbreviations: CC, caregiver contribution; CC‐SC‐CII, caregiver contribution to self‐care of chronic illness inventory; CSE‐CSC, caregiver self‐efficacy in contributing to self‐care scale; ITC, item‐total correlation coefficient; ku, kurtosis; SD, standard deviation; sk, skewness.

Across all scales and subscales, no inter‐item correlation was below the recommended lower threshold of 0.30. For the CC‐SC‐CII maintenance scale, inter‐item correlations ranged from 0.40 to 0.76, with 4/21 pairs exceeding 0.70. For the monitoring scale, correlations ranged from 0.61 to 0.78, with 6/10 pairs above 0.70. For the management scale, correlations ranged from 0.46 to 0.86, with 4/15 pairs above 0.70. For the CSE‐CSC, inter‐item correlations ranged from 0.37 to 0.83, with 10/45 pairs above 0.70. Corrected item‐total correlations were all above 0.30 across the CC‐SC‐CII maintenance (0.57–0.81), monitoring (0.73–0.84), management (0.67–0.82), and CSE‐CSC (0.52–0.82) scales.

### 3.3 Structural Validity

#### 3.3.1 Caregiver Contribution to Self‐Care of Chronic Illness Inventory

**FIGURE 1:**
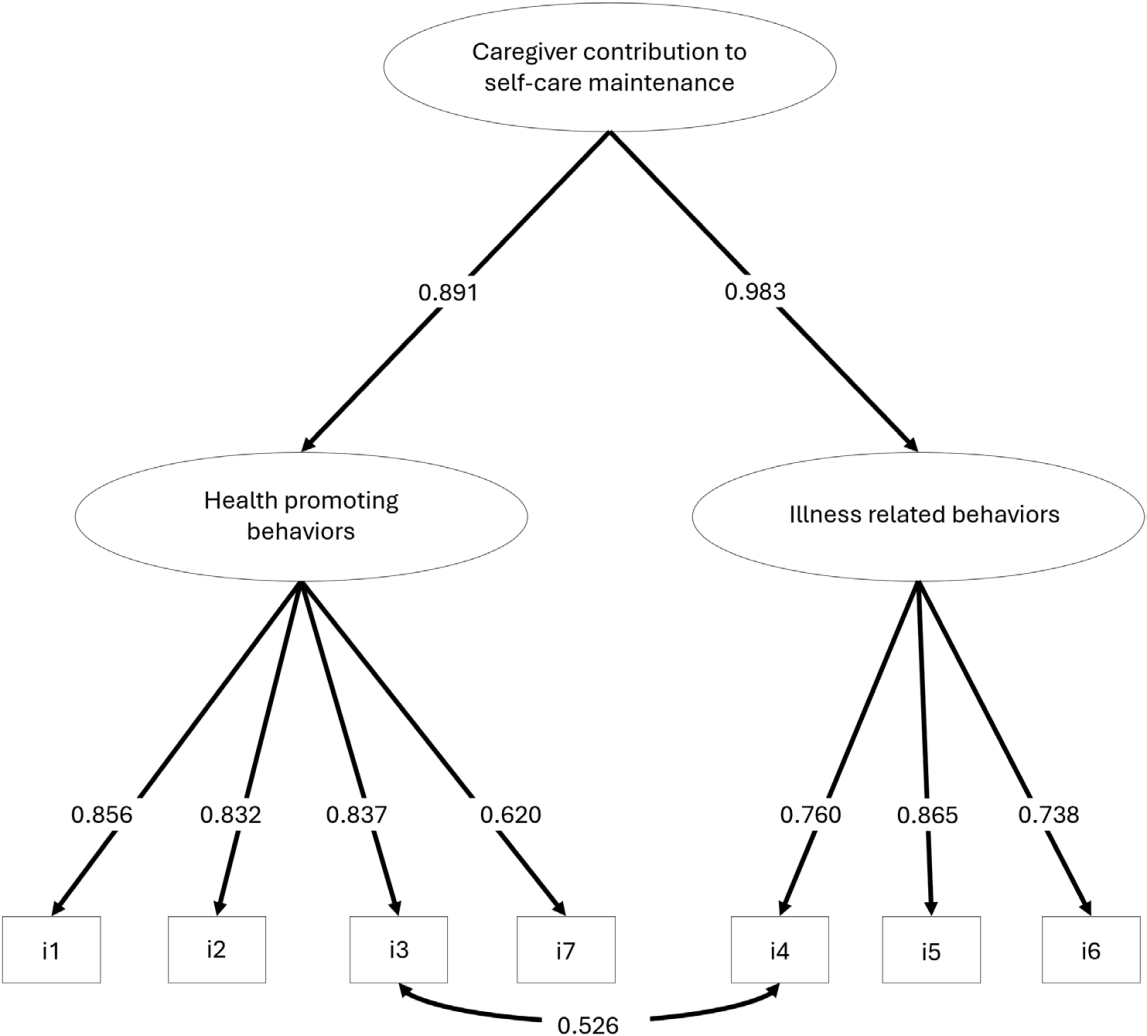
Confirmatory factor analysis of the caregiver contribution to self‐care maintenance scale. One‐headed arrows show factor loadings of individual items on the latent construct, while double‐headed arrows indicate error covariances. The completely standardized solution was generated using Mplus.

The CFA conducted on the CC to self‐care maintenance scale was specified with the two factors of health promoting behaviors (items #1, #3, and #7) and illness‐related behaviors (items #2, #4, #5, and #6). This model yielded an acceptable fit, except for RMSEA: *χ* ^2^ (13, *N* = 409) = 65.84, *p* < 0.001, CFI = 0.94, TLI = 0.90, RMSEA = 0.100 (90% CI = 0.077–0.124), *p* < 0.001, SRMR = 0.039. Inspection of the modification indices suggested that item #2 should load onto the health promoting behaviors and that items #3 and #4 should covary. Accordingly, the model was respecified accommodating these modifications, resulting in a dramatic improvement in fit: *χ* ^2^ (12, *N* = 409) = 14.22, *p* = 0.29, CFI = 1.00, TLI = 1.00, RMSEA = 0.021 (90% CI = 0.000–0.057), *p* = 0.895, SRMR = 0.020. Since the two factors were highly correlated (*r* = 0.88) and in line with the Middle Range of self‐care theory, a second‐order factor was specified, which also yielded a satisfactory fit: *χ* ^2^ (11, *N* = 409) = 13.45, *p* = 0.26, CFI = 1.00, TLI = 0.99, RMSEA = 0.023 (90% CI = 0.000–0.060), *p* = 0.868, SRMR = 0.020. All factor loadings were high and statistically significant (Figure 1).

**FIGURE 2:**
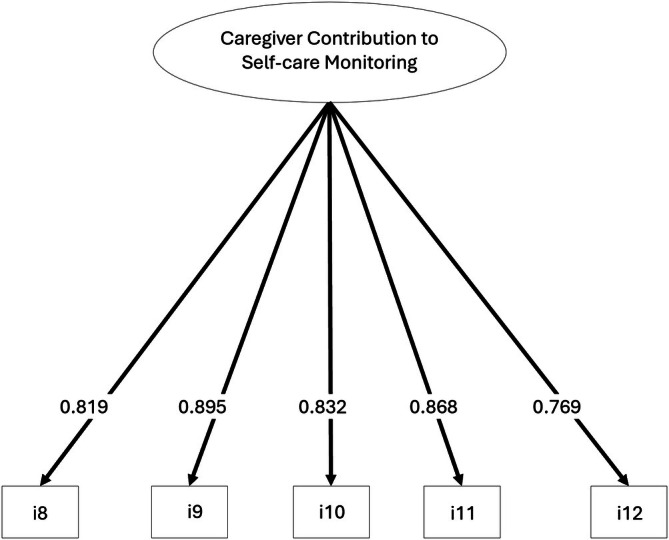
Confirmatory factor analysis of the caregiver contribution to self‐care monitoring scale. One‐headed arrows show factor loadings of individual items on the latent construct. The completely standardized solution was generated using Mplus.

The CFA conducted on the CC to self‐care monitoring scale was specified as a single factor comprising the items #8, #9, 10, #11, and #12. This model yielded an excellent fit: *χ* ^2^ (5, *N* = 409) = 7.09, *p* = 0.21, CFI = 1.00, TLI = 0.99, RMSEA = 0.032 (90% CI = 0.000–0.081), *p* = 0.666, SRMR = 0.017. All factor loadings were high and significant (Figure 2).

**FIGURE 3:**
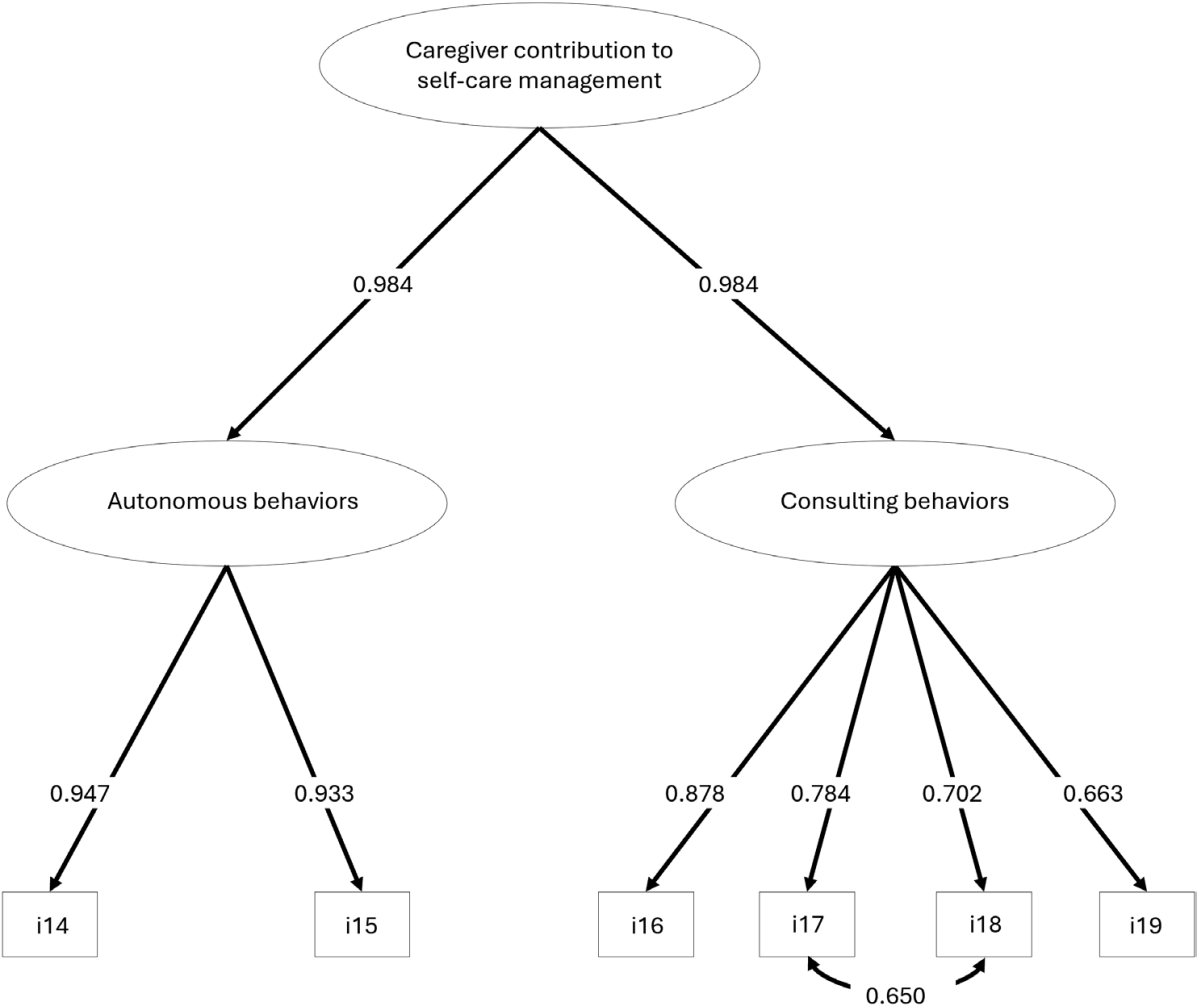
Confirmatory factor analysis of the caregiver contribution to self‐care management scale. One‐headed arrows show factor loadings of individual items on the latent construct, while double‐headed arrows indicate error covariances. The completely standardized solution was generated using Mplus.

The CFA conducted on the CC to self‐care management scale was specified with the two factors of autonomous behaviors (items #14 and #15) and consulting behaviors (items #16, #17, #18, and #19) as in previous Italian psychometric validation (Vellone, Lorini, et al. 2020). This model yielded a poor fit: χ^2^ (8, *N* = 394) = 99.44, *p* < 0.001, CFI = 0.91, TLI = 0.84, RMSEA = 0.170 (90% CI = 0.141–0.201), *p* < 0.001, SRMR = 0.050. Examination of the modification indices indicated that the misfit was caused by an excessive covariation between item #17 and #18. After allowing the residuals of these two items to freely covariate, the fit was excellent: *χ* ^2^ (8, *N* = 394) = 10.89, *p* = 0.144, CFI = 1.00, TLI = 0.99, RMSEA = 0.038 (90% CI = 0.000–0.078), *p* = 0.639, SRMR = 0.020. Since the two factors were highly correlated (*r* = 0.79) and in line with the Middle Range of self‐care theory, we also specified a second‐order factor which yielded a satisfactory fit: *χ* ^2^ (7, *N* = 394) = 11.28, *p* = 0.12, CFI = 1.00, TLI = 0.99, RMSEA = 0.039 (90% CI = 0.000–0.080), *p* = 0.613, SRMR = 0.020. All factor loadings were high and statistically significant (Figure 3).

#### 3.3.2 Caregiver Self‐Efficacy in Contributing to Patient Self‐Care

**FIGURE 4:**
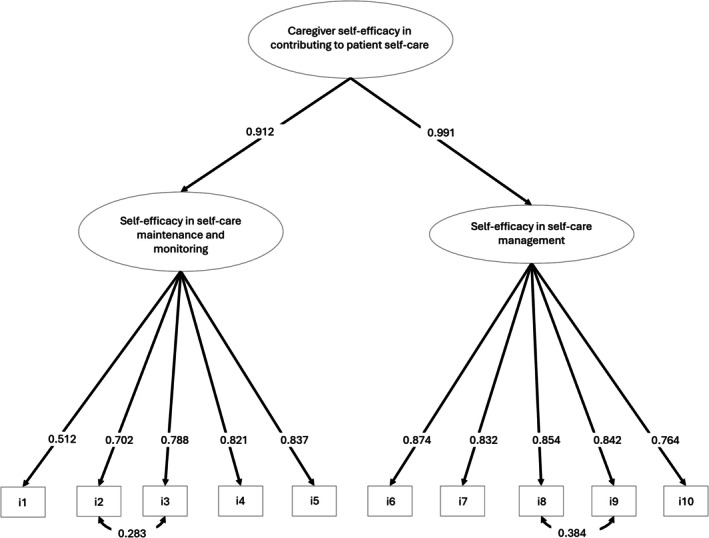
Confirmatory factor analysis of the caregiver self‐efficacy in contributing to patient self‐care (CSE‐CSC). One‐headed arrows show factor loadings of individual items on the latent construct, while double‐headed arrows indicate error covariances. The completely standardized solution was generated using Mplus.

The CFA conducted on the CSE‐CSC scale was initially specified as a single‐factor model, consistent with previous findings (Di Nitto et al. 2025). However, this model yielded a poor fit: *χ* ^2^ (35, *N* = 409) = 159.16, *p* < 0.001, CFI = 0.87, TLI = 0.83, RMSEA = 0.093 (90% CI = 0.079–0.108), *p* < 0.001, SRMR = 0.065. Although modification indices suggested several covariances that could improve model fit, these adjustments were not theoretically justifiable. Thus, we specified a two‐factor model, consistent with the original factorial structure of the scale (De Maria et al. 2021): self‐efficacy in self‐care maintenance and monitoring (item #1, #2, #3, #4, and #5) and self‐efficacy in self‐care management (#6, #7, #8, #9, and #10). This solution yielded a marginal fit: *χ* ^2^ (34, *N* = 409) = 121.30, *p* < 0.001, CFI = 0.91, TLI = 0.88, RMSEA = 0.079 (90% CI = 0.064–0.095), *p* = 0.001, SRMR = 0.059. Inspection of the modification indices revealed high values for the covariances between items #2 and #3, and items #8 and #9. After specifying these covariances, the model fit improved: *χ* ^2^ (32, *N* = 409) = 103.64, *p* < 0.001, CFI = 0.93, TLI = 0.90, RMSEA = 0.074 (90% CI = 0.058–0.090), *p* = 0.007, SRMR = 0.051. Since the two factors were highly correlated (*r* = 0.90), we also specified a second‐order factor which yielded an acceptable fit: *χ* ^2^ (31, *N* = 409) = 127.88, *p* = < 0.001, CFI = 0.92, TLI = 0.90, RMSEA = 0.087 (90% CI = 0.072–0.103), *p* < 0.001, SRMR = 0.051 (Table 4). All factor loadings were high and statistically significant (Figure 4).

**TABLE 4:** Summary of confirmatory factor analysis fit indices for the CC‐SC‐CII and CSE‐CSC scales.

Scale/subscale	*χ* ^2^ (df, *p*)	CFI	TLI	RMSEA (90% CI)	SRMR
CC‐SC‐CII—Maintenance	13.45 (11, 0.26)	1.00	1.00	0.023 (0.000–0.060)	0.020
CC‐SC‐CII—Monitoring	7.09 (5, 0.21)	1.00	0.99	0.032 (0.000–0.081)	0.017
CC‐SC‐CII—Management	11.28 (7, 0.12)	1.00	0.99	0.039 (0.000–0.080)	0.020
CSE‐CSC	127.88 (31, < 0.001)	0.92	0.90	0.087 (0.072–0.103)	0.051

Abbreviations: CFI, Comparative Fit Index; df, degrees of freedom; *p*, *p*‐value; RMSEA, Root Mean Square Error of Approximation; SRMR, Standardized Root Mean Square Residual; TLI, Tucker‐Lewis Index.

### 3.4 Reliability

#### 3.4.1 Caregiver Contribution to Self‐Care of Chronic Illness Inventory

The internal consistency reliability of the CC to self‐care maintenance scale was optimal, with factor determinacy scores of 0.95 and 0.94 for the health‐promoting and illness‐related behaviors subscales, respectively. The Omega coefficients were also adequate for these subscales, at 0.88 and 0.83, respectively. The factor determinacy score and omega coefficient for the CC to self‐care monitoring scale were 0.96 and 0.92, respectively. The internal consistency reliability of the CC to self‐care management scale was also optimal, with factor determinacy scores of 0.98 for the autonomous and consulting behaviors subscales. The Omega coefficients for these subscales were likewise adequate at 0.89 and 0.90, respectively. The ICCs between enrolment and T1 (3 months after enrolment) were adequate for CC to self‐care maintenance (ICC 0.772 = 95% CI: 0.727–0.811) and CC to self‐care monitoring (ICC 0.795 = 95% CI: 0.753–0.830), while it was slightly below the threshold for self‐care management (ICC 0.644 = 95% CI: 0.580–0.701). Lastly, all the items indicated that caregivers frequently contributed to self‐care and had high levels of confidence in their behaviors. Item total correlations were all above 0.30 for all subscales, indicating all items were appropriate.

#### 3.4.2 Caregiver Self‐Efficacy in Contributing to Patient Self‐Care

The internal consistency reliability of the CSE‐CSC scale was adequate, with factor determinacy scores of 0.95 and 0.96 for the factors self‐efficacy in self‐care maintenance and monitoring, and self‐efficacy in self‐care management, respectively. The Omega coefficients were also adequate for the same subscales at 0.85 and 0.93, respectively. ICC between enrolment and T1 (3 months after enrolment) was adequate (ICC 0.708 = 95% CI: 0.652–0.756).

### 3.5 Construct Validity

CC to self‐care maintenance showed a non‐significant correlation with patient self‐care maintenance (*r* = 0.14; *p* = 0.212), CC to self‐care monitoring showed a positive weak correlation with patient's self‐care monitoring (*r* = 0.21; *p* < 0.001), and CC to self‐care management showed a positive moderate correlation with patient self‐care management (*r* = 0.43; *p* < 0.001). Lastly, all three CC‐SC‐CII subscales and the CSE‐CSC were significantly correlated (maintenance: *r* = 0.18, *p* = 0.002; monitoring: *r* = 0.41, *p* < 0.001; management: *r* = 0.32, *p* < 0.001) (Table 5).

**TABLE 5:** Correlations among the CC‐SC‐CII and CSE‐CSC, and other variables.

	CC to self‐care maintenance	CC to self‐care monitoring	CC to self‐care management	CSE‐CSC
Self‐care maintenance	0.14 (0.212)	—	—	0.18 (0.009)
Self‐care monitoring	—	0.21 (< 0.001)	—	0.41 (< 0.001)
Self‐care management	—	—	0.43 (< 0.001)	0.32 (< 0.001)

*Note:* Pearson's *r* and *p*‐values in parenthesis are shown.

Abbreviations: CC‐SC‐CII, caregiver contribution to self‐care of chronic illness inventory; CSE‐CSC, caregiver self‐efficacy in contributing to self‐care scale; SC‐SE, self‐care self‐efficacy; T1, time 1, 3 months after enrolment.

## 4 Discussion

This study examined the psychometric properties of two instruments assessing CC and self‐efficacy in caregivers of patients with cancer. To our knowledge, it is the first validation of these measures in an oncological caregiving context. The results confirm that both scales are reliable and valid for evaluating caregiver behaviors and confidence, supporting their use in oncology to measure CC to self‐care and self‐efficacy.

The dimensional structure of the CC scale mirrored previous validations in chronic disease populations (Adëraj et al. 2025; Vellone, Lorini, et al. 2020). Factor loadings across all subscales were significant, confirming that items effectively represent the construct in caregivers of cancer patients.

Covariance between specific CC to self‐care maintenance items (#3 “physical activity” and #4 “eat special foods”) likely reflects semantic proximity and the behavioral link between physical activity and healthy diet practices caregivers often encourage to mitigate chemotherapy side effects (Sollazzo et al. 2023) and improve treatment effectiveness (Rock et al. 2020).

For the monitoring scale, confirmatory factor analysis supported a one‐factor model consistent with an earlier study (Adëraj et al. 2025). In the management scale, the covariance freed between items addressing communication with healthcare providers (#17 and #18) improved model fit, as found in a previous study (Adëraj et al. 2025). These items both reflect consultation behaviors, suggesting that caregivers perceive contacting and informing healthcare providers as related actions aimed at evaluating treatment effectiveness. For the self‐efficacy scale, covariances between items addressing treatment adherence (#2 and #3) and symptom management (#8 and #9) were justified by similar conceptual content and item proximity (Weijters et al. 2009).

At the scale score level, ceiling effects were observed for the CC‐SC‐CII maintenance, monitoring, and management scales, particularly for monitoring (58.92%). At the item level, high endorsement of the highest response category was common for several CC‐SC‐CII items, indicating frequent caregiver involvement in self‐care activities, especially symptom monitoring and treatment‐related surveillance. This pattern is clinically plausible in caregivers of patients receiving oral anticancer agents, as previous qualitative evidence shows that caregivers play a central role in monitoring symptoms, managing treatment‐related problems, and supporting home‐based self‐care (Sollazzo et al. 2023). Some inter‐item correlations exceeded 0.70, suggesting conceptual proximity among selected items. However, no correlation was below 0.30, all corrected item‐total correlations were acceptable, and CFA supported the expected dimensional structure. Thus, these findings do not undermine the reliability or factorial validity of the instruments, but suggest that the CC‐SC‐CII may have reduced ability to discriminate among caregivers with very high contribution levels. By contrast, the CSE‐CSC did not exceed either the scale‐level or item‐level ceiling‐effect thresholds. Upper end scores should therefore be interpreted cautiously, particularly when evaluating change over time or intervention effects (Terwee et al. 2007).

Construct validity was only partially supported: no significant correlation emerged between caregiver and patient self‐care maintenance, differing from earlier studies in chronic conditions (Fabrizi et al. 2025; Vellone, Lorini, et al. 2020). This difference may reflect the oncology‐specific distribution of caregiving roles, rather than a general weakness of the construct. Specifically, in the context of cancer care, particularly among patients receiving OAAs, self‐care includes medication adherence and preventive behaviors, but also symptom surveillance, recognition of illness changes, side‐effect management, and decisions about when to seek professional support (Di Nitto et al. 2022). Therefore, caregivers may be more actively involved in monitoring and management behaviors than in routine maintenance behaviors, especially when treatment demands and symptom burden fluctuate. This interpretation is also consistent with qualitative evidence showing that caregivers of patients treated with OAAs contribute substantially to tracking symptoms and side effects and to managing worsening symptoms (Sollazzo et al. 2023).

Conversely, CC to self‐care monitoring showed a modest positive correlation with patient monitoring (*r* = 0.21; *p* < 0.001), aligning with previous evidence (Vellone, Lorini, et al. 2020). The concept of caregiving bind (Moore and Gillespie 2014) may explain this weaker association: patients might underreport symptoms to avoid burdening caregivers, reducing observed congruence (Filipponi et al. 2023). This dynamic may result in attenuated statistical correlation despite actual caregiving involvement. Lastly, CC to self‐care management correlated moderately with patient management (*r* = 0.43; *p* < 0.001), consistent with prior studies (Adëraj et al. 2025; Vellone, Lorini, et al. 2020) and likely reflecting the shared nature of decision‐making in cancer symptom control, whereby patients and caregivers jointly evaluate symptoms, decide when to implement self‐care strategies, and determine when professional support is needed (Cerutti et al. 2025).

All CC to self‐care scales correlated significantly with self‐efficacy, confirming that greater confidence enhances contribution (De Maria et al. 2021), although the association between self‐efficacy and CC to self‐care maintenance was weaker than expected. Internal consistency was strong across scales (Omega > 0.80), and test–retest reliability was acceptable (ICC > 0.70) except for the monitoring scale (ICC = 0.64). The lower stability likely reflects the relatively long three‐month test–retest interval, combined with variability in the cancer trajectory in terms of physical health and functioning (Bellizzi et al. 2024). Over this period, changes in patients' clinical conditions and treatment demands may lead to genuine variations in caregiver behaviors and perceived self‐efficacy, rather than measurement error alone. As patients' conditions fluctuate, caregiver involvement in monitoring may increase during deterioration and decrease in stable phases, influencing reliability outcomes. Similar patterns have been reported in dyads managing multiple chronic illnesses (Iovino et al. 2021), supporting the view that CC varies dynamically with patient health status. Therefore, test–retest estimates should be interpreted as reflecting stability of the instruments over a clinically meaningful interval within a dynamic care context, rather than as strict short‐term test–retest reliability.

### 4.1 Limitations

Some limitations must be acknowledged. First, the use of a convenience sample may limit the external validity of the findings, as participants may not be representative of the broader population of caregivers for patients with cancer. An additional aspect to consider is the recruitment setting. Although caregivers were enrolled in outpatient contexts, data collection occurred during clinical visits, which may have temporarily influenced caregivers' perceptions of their role and confidence due to interaction with healthcare professionals. However, the CC‐SC‐CII refers to behaviors over the previous month and the CSE‐CSC refers to self‐efficacy perception in general, thus referring to the home environment, as the place where the caregivers mostly spend time in contributing to self‐care behaviors for patients receiving oral anticancer agents. Therefore, the impact of the recruitment setting on the observed psychometric properties is likely to be limited. Second, reliability over time was assessed using a three‐month test–retest interval, which is longer than the intervals commonly recommended for evaluating strict temporal stability. The choice of a three‐month interval was driven by the longitudinal design of the study and to avoid excessive burden for caregivers. However, future research should consider shorter retest intervals (1 month) to better isolate the temporal stability of these instruments. Third, the CFA models included a few theoretically justified residual covariances suggested by modification indices. Although this approach is accepted in psychometric modeling, future studies should cross‐validate these factor structures in independent samples to further reduce the risk of capitalization on chance.

Fourth, the construct validity of the CC to self‐care maintenance with the patient self‐care maintenance was not confirmed, and future studies should explore this aspect. Lastly, the study was conducted within a single national context, and cultural factors potentially influencing caregiving practices were not examined. Therefore, measurement invariance of the CC‐SC‐CII and CSE‐CSC scales remains to be established.

## 5 Conclusion

This study confirmed that the CC‐SC‐CII and CSE‐CSC are reliable and valid instruments for assessing caregiver support for self‐care and self‐efficacy in the context of cancer disease. Both tools demonstrated strong measurement properties and can assist nurses and healthcare professionals in understanding the caregiver's role, identifying areas where additional support is needed, and designing tailored interventions. Their use can facilitate the identification of areas where caregivers may require additional support and inform the planning of tailored, nurse‐led interventions.

### 5.1 Relevance for Clinical Practice

This study strengthens evidence supporting assessment of CC to self‐care in cancer care contexts requiring ongoing home‐based self‐care (e.g., during active treatment or survivorship). The psychometric validation of the CC‐SC‐CII and CSE‐CSC provides reliable tools for evaluating caregiver behaviors and confidence in self‐care. Because the instruments are grounded in a generic chronic‐illness self‐care framework, they may provide a useful model for assessing CC and caregiver self‐efficacy in other long‐term caregiving contexts, including high‐demand CC such as dementia. However, additional psychometric validation is needed before routine use in caregiver populations beyond oncology or those in which the instruments have already been validated. In clinical practice, these instruments may support nurses in systematically identifying caregivers who report lower contribution or self‐efficacy and in tailoring educational or supportive strategies accordingly. In practice, these instruments may be used to screen caregivers for lower levels of contribution to self‐care or self‐efficacy, supporting early identification of caregivers who may require additional support. Assessment results can inform individualized care planning, guiding nurses in tailoring educational and supportive interventions to caregivers' specific needs. While the use of these tools does not imply direct improvement in patient or caregiver outcomes, they can inform clinical assessment, enhance patient–caregiver–nurse communication, and support research on caregiver trajectories, intervention development, and cross‐condition comparisons.

Future studies should examine how dyadic dynamics influence caregiver and patient self‐care patterns and identify typologies of caregiver‐patient relationships in oncology.

## Author Contributions

**Vincenzo Damico:** investigation, writing – review and editing. **Marco Di Nitto:** conceptualization, data curation, formal analysis, project administration, writing – original draft. **Federica Lacarbonara:** writing – original draft. **Sipontina Rita Zerulo:** investigation, writing – review and editing. **Paolo Iovino:** formal analysis, writing – original draft. **Angela Durante:** writing – review and editing.

## Funding

This research was funded by the Centre of Excellence for Nursing Scholarship (CECRI), Rome. The founder did not influence the study design, data collection, analysis, interpretation, or report writing. The vision expressed in this paper is the author's and does not represent any involvement of the bodies or authorities of affiliation.

## Ethics Statement

This study adhered to the Good Clinical Practice Standards of the European Union and the Helsinki Declaration. Before starting data collection, this study was approved by the Ethics Committee of the “Policlinico Tor Vergata of Rome” on 20/09/2022 (reference number 188.22). Participation was voluntary; all participants were fully informed about the study aims and could withdraw from the study at any time. Enrolment occurred only after participants had signed the informed consent form.

## Conflicts of Interest

The authors declare no conflicts of interest.

## Supporting information


**Supporting Information: S1.** General reporting recommendations relevant for all studies on measurement properties.

## Data Availability

The data that support the findings of this study are available from the corresponding author upon reasonable request.

## References

[nhs70380-bib-0001] Adëraj, S. , A. Arapi , R. Mazzotta , et al. 2025. “Caregiver Contribution to Self‐Care of Chronic Illness Inventory: Evaluation of Measurement Properties in a Middle‐Income Country.” Nursing Reports 15, no. 2: 42. 10.3390/nursrep15020042. 39997777 PMC11858183

[nhs70380-bib-0002] Ahmad, T. A. , D. P. Gopal , C. Chelala , A. Z. Dayem Ullah , and S. J. Taylor . 2023. “Multimorbidity in People Living With and Beyond Cancer: A Scoping Review.” American Journal of Cancer Research 13, no. 9: 4346–4365. 37818046 PMC10560952

[nhs70380-bib-0003] Alavi, M. , D. C. Visentin , D. K. Thapa , G. E. Hunt , R. Watson , and M. Cleary . 2020. “Chi‐Square for Model Fit in Confirmatory Factor Analysis.” Journal of Advanced Nursing 76, no. 9: 2209–2211. 10.1111/jan.14399. 32323338

[nhs70380-bib-0004] Bellizzi, K. M. , C. L. Park , J. W. Lee , et al. 2024. “Physical Health and Function Trajectories in Adults With Cancer: Psychosocial Predictors of Class Membership.” Journal of Cancer Survivorship 19: 1173–1183. 10.1007/s11764-024-01540-3. 38289507 PMC11286833

[nhs70380-bib-0005] Bradley, C. J. 2019. “Economic Burden Associated With Cancer Caregiving.” Seminars in Oncology Nursing 35, no. 4: 333–336. 10.1016/j.soncn.2019.06.003. 31229344 PMC6660380

[nhs70380-bib-0006] Brown, T. A. 2015. Confirmatory Factor Analysis for Applied Research. 2nd ed. Guilford Publications.

[nhs70380-bib-0007] Caggianelli, G. , F. Alivernini , A. Chirico , et al. 2024. “The Relationship Between Caregiver Contribution to Self‐Care and Patient Quality of Life in Heart Failure: A Longitudinal Mediation Analysis.” PLoS One 19, no. 3: e0300101. 10.1371/journal.pone.0300101. 38470867 PMC10931462

[nhs70380-bib-0008] Castro, A. R. , A. Arnaert , K. Moffatt , J. Kildea , V. Bitzas , and A. Tsimicalis . 2023. ““Informal Caregiver” in Nursing: An Evolutionary Concept Analysis.” Advances in Nursing Science 46, no. 1: E29–E42. 36006014 10.1097/ANS.0000000000000439

[nhs70380-bib-0009] Cerutti, J. , M. C. Lent , R. F. Holcombe , and M. Reblin . 2025. “Patient and Caregiver Perceptions of Caregiving Contributions During Cancer Clinical Trials: A Mixed‐Methods Study.” Cancer Medicine 14, no. 1: e70488. 10.1002/cam4.70488. 39781576 PMC11712184

[nhs70380-bib-0010] De Maria, M. , P. Iovino , S. Lorini , D. Ausili , M. Matarese , and E. Vellone . 2021. “Development and Psychometric Testing of the Caregiver Self‐Efficacy in Contributing to Patient Self‐Care Scale.” Value in Health 24, no. 10: 1407–1415. 10.1016/j.jval.2021.05.003. 34593163

[nhs70380-bib-0011] Di Nitto, M. , A. Durante , F. Torino , et al. 2025. “Validity and Reliability of the Self‐Care of Chronic Illness Inventory and Self‐Care Self‐Efficacy Scale in Patients Living With Cancer.” Journal of Advanced Nursing 81: 8620–8632. 10.1111/jan.16823. 39968728 PMC12623676

[nhs70380-bib-0012] Di Nitto, M. , F. Sollazzo , V. Biagioli , et al. 2022. “Self‐Care Behaviours in Older Adults Treated With Oral Anticancer Agents: A Qualitative Descriptive Study.” European Journal of Oncology Nursing 58: 102139. 10.1016/j.ejon.2022.102139. 35489295

[nhs70380-bib-0013] Erba, I. , M. De Maria , M. Saurini , D. Ausili , M. Matarese , and E. Vellone . 2025. “Generic and Disease‐Specific Caregiver Contribution to Self‐Care in a Population With Multiple Chronic Conditions: A Comparative Study.” Journal of Clinical Nursing 34, no. 5: 1787–1800. 10.1111/jocn.17334. 38951119

[nhs70380-bib-0014] Fabrizi, D. , M. De Maria , C. Barbaranelli , et al. 2025. “Development and Psychometric Testing of the Caregiver Contribution to Self‐Care of Diabetes Inventory: An Observational Study Among Informal Caregivers of Patients With Type 2 Diabetes.” Science of Diabetes Self Management and Care 51, no. 3: 281–300. 10.1177/26350106251336309. 40411395

[nhs70380-bib-0015] Filipponi, C. , M. Chichua , M. Masiero , D. Mazzoni , and G. Pravettoni . 2023. “Cancer Pain Experience Through the Lens of Patients and Caregivers: Mixed Methods Social Media Study.” JMIR Cancer 9: e41594. 10.2196/41594. 37399067 PMC10365594

[nhs70380-bib-0016] Gagnier, J. J. , J. Lai , L. B. Mokkink , and C. B. Terwee . 2021. “COSMIN Reporting Guideline for Studies on Measurement Properties of Patient‐Reported Outcome Measures.” Quality of Life Research 30, no. 8: 2197–2218. 10.1007/s11136-021-02822-4. 33818733

[nhs70380-bib-0017] Hu, L. , and P. M. Bentler . 1999. “Cutoff Criteria for Fit Indexes in Covariance Structure Analysis: Conventional Criteria Versus New Alternatives.” Structural Equation Modeling: A Multidisciplinary Journal 6, no. 1: 1–55. 10.1080/10705519909540118.

[nhs70380-bib-0018] Iovino, P. , K. S. Lyons , M. De Maria , et al. 2021. “Patient and Caregiver Contributions to Self‐Care in Multiple Chronic Conditions: A Multilevel Modelling Analysis.” International Journal of Nursing Studies 116: 103574. 10.1016/j.ijnurstu.2020.103574. 32276720

[nhs70380-bib-0019] Kline, R. 2023. Principles and Practices of Structural Equation Modeling. 5th ed. Guilford Press.

[nhs70380-bib-0020] Korkmaz, S. , D. Goksuluk , and G. Zararsiz . 2014. “MVN: An R Package for Assessing Multivariate Normality.” R Journal 6, no. 2: 151–162. 10.32614/RJ-2014-031.

[nhs70380-bib-0021] Lee, J. , K. Kim , E. Vellone , and S. Park . 2025. “Caregiver Self‐Efficacy in Contributing to Patient Self‐Care (CSE‐CSC) Scale: Psychometric Testing in a Population of Caregivers of Parkinson's Disease in the Republic of Korea.” Applied Nursing Research 84: 151981. 10.1016/j.apnr.2025.151981. 40592654

[nhs70380-bib-0022] Lv, X. , W. Ren , S. Ran , et al. 2023. “Trends and Prescribing Patterns of Oral Anti‐Neoplastic Drugs: A Retrospective Longitudinal Study.” Frontiers in Public Health 11: 1294126. 10.3389/fpubh.2023.1294126. 38074729 PMC10701268

[nhs70380-bib-0023] Matarese, M. , R. Pendoni , D. Ausili , E. Vellone , and M. De Maria . 2023. “Validity and Reliability of Caregiver Contribution to Self‐Care of Chronic Obstructive Pulmonary Disease Inventory and Caregiver Self‐Efficacy in Contributing to Self‐Care Scale.” Evaluation & the Health Professions 46, no. 3: 255–269. 10.1177/01632787221134712. 36266087

[nhs70380-bib-0024] McDonald, R. P. 1999. Test Theory: A Unified Treatment. 1st ed. Psychology Press.

[nhs70380-bib-0025] Moore, H. , and A. Gillespie . 2014. “The Caregiving Bind: Concealing the Demands of Informal Care Can Undermine the Caregiving Identity.” Social Science & Medicine 116: 102–109. 10.1016/j.socscimed.2014.06.038. 24996218

[nhs70380-bib-0026] Muthén, L. K. , and B. Muthén . 2017. MPlus Statistical Analysis With Latent Variables: User's Guide. Muthén & Muthén.

[nhs70380-bib-0027] Polit, D. , and F. Yang . 2016. Measurement and the Measurement of Change: A Primer for the Health Professions. Wolters Kluwer Health.

[nhs70380-bib-0028] Riegel, B. , T. Jaarsma , and A. Strömberg . 2012. “A Middle‐Range Theory of Self‐Care of Chronic Illness.” Advances in Nursing Science 35, no. 3: 194–204. 22739426 10.1097/ANS.0b013e318261b1ba

[nhs70380-bib-0029] Rock, C. L. , C. Thomson , T. Gansler , et al. 2020. “American Cancer Society Guideline for Diet and Physical Activity for Cancer Prevention.” CA: A Cancer Journal for Clinicians 70, no. 4: 245–271. 10.3322/caac.21591. 32515498

[nhs70380-bib-0030] Sollazzo, F. , M. Di Nitto , L. Rosito , et al. 2023. “Caregivers' Contribution to Self‐Care for Patients Treated With Oral Anticancer Agents: A Qualitative Descriptive Study.” European Journal of Oncology Nursing 64: 102327. 10.1016/j.ejon.2023.102327. 37156057

[nhs70380-bib-0031] Terwee, C. B. , S. D. Bot , M. R. de Boer , et al. 2007. “Quality Criteria Were Proposed for Measurement Properties of Health Status Questionnaires.” Journal of Clinical Epidemiology 60, no. 1: 34–42. 10.1016/j.jclinepi.2006.03.012. 17161752

[nhs70380-bib-0032] Vellone, E. , V. Biagioli , A. Durante , et al. 2020. “The Influence of Caregiver Preparedness on Caregiver Contributions to Self‐Care in Heart Failure and the Mediating Role of Caregiver Confidence.” Journal of Cardiovascular Nursing 35, no. 3: 243–252. 10.1097/jcn.0000000000000632. 32084078

[nhs70380-bib-0033] Vellone, E. , S. Lorini , D. Ausili , et al. 2020. “Psychometric Characteristics of the Caregiver Contribution to Self‐Care of Chronic Illness Inventory.” Journal of Advanced Nursing 76, no. 9: 2434–2445. 10.1111/jan.14448. 32538503

[nhs70380-bib-0034] Vellone, E. , B. Riegel , and R. Alvaro . 2019. “A Situation‐Specific Theory of Caregiver Contributions to Heart Failure Self‐Care.” Journal of Cardiovascular Nursing 34, no. 2: 166–173. 10.1097/jcn.0000000000000549. 30363017

[nhs70380-bib-0035] Weijters, B. , M. Geuens , and N. Schillewaert . 2009. “The Proximity Effect: The Role of Inter‐Item Distance on Reverse‐Item Bias.” International Journal of Research in Marketing 26, no. 1: 2–12. 10.1016/j.ijresmar.2008.09.003.

